# Predictors of Current and Longer-Term Patterns of Abundance of American Pikas (*Ochotona princeps*) across a Leading-Edge Protected Area

**DOI:** 10.1371/journal.pone.0167051

**Published:** 2016-11-30

**Authors:** Lucas Moyer-Horner, Erik A. Beever, Douglas H. Johnson, Mark Biel, Jami Belt

**Affiliations:** 1 Department of Biology, University of Utah, Salt Lake City, Utah, United States of America; 2 U.S. Geological Survey, Northern Rocky Mountain Science Center, Bozeman, Montana, United States of America; 3 Department of Ecology, Montana State University, Bozeman, Montana, United States of America; 4 U.S. Geological Survey, Northern Prairie Wildlife Research Center, Saint Paul, Minnesota, United States of America; 5 Fisheries, Wildlife, and Conservation Biology Department, University of Minnesota, Saint Paul, Minnesota, United States of America; 6 Glacier National Park, National Park Service, West Glacier, Montana, United States of America; 7 Klondike Gold Rush National Historical Park, National Park Service, Skagway, Alaska, United States of America; Università degli Studi di Napoli Federico II, ITALY

## Abstract

American pikas (*Ochotona princeps*) have been heralded as indicators of montane-mammal response to contemporary climate change. Pikas no longer occupy the driest and lowest-elevation sites in numerous parts of their geographic range. Conversely, pikas have exhibited higher rates of occupancy and persistence in Rocky Mountain and Sierra Nevada montane ‘mainlands’. Research and monitoring efforts on pikas across the western USA have collectively shown the nuance and complexity with which climate will often act on species in diverse topographic and climatic contexts. However, to date no studies have investigated habitat, distribution, and abundance of pikas across hundreds of sites within a remote wilderness area. Additionally, relatively little is known about whether climate acts most strongly on pikas through direct or indirect (e.g., vegetation-mediated) mechanisms. During 2007–2009, we collectively hiked >16,000 km throughout the 410,077-ha Glacier National Park, Montana, USA, in an effort to identify topographic, microrefugial, and vegetative characteristics predictive of pika abundance. We identified 411 apparently pika-suitable habitat patches with binoculars (*in situ*), and surveyed 314 of them for pika signs. Ranking of alternative logistic-regression models based on AIC_*c*_ scores revealed that short-term pika abundances were positively associated with intermediate elevations, greater cover of mosses, and taller forbs, and decreased each year, for a total decline of 68% during the three-year study; whereas longer-term abundances were associated only with static variables (longitude, elevation, gradient) and were lower on north-facing slopes. Earlier Julian date and time of day of the survey (i.e., midday vs. not) were associated with lower observed pika abundance. We recommend that wildlife monitoring account for this seasonal and diel variation when surveying pikas. Broad-scale information on status and abundance determinants of montane mammals, especially for remote protected areas, is crucial for land and wildlife-resource managers trying to anticipate mammalian responses to climate change.

## Introduction

One of the primary challenges to effective wildlife conservation in the 21^st^ century involves predicting the direct and indirect effects of climate change [1,2]. Accurate broad-scale predictions are difficult to obtain because future climates will: likely be novel [3], and exhibit magnitudes of change that vary markedly across the globe, ecoregions, and even in small, topographically complex areas (e.g., [4,5,6]). Analogously, multi-species predictions are complicated by observations that species have tended to respond individualistically to the complex effects of climate change over paleontological and ecological timescales (e.g., [7,8,9,10,11]. In addition, the ability to identify widespread patterns has been limited by a dearth of biogeographic data on the local and regional distributions and abundance of many species [12,13]. Effective species conservation requires a better understanding of the environmental factors and mechanisms that drive changes in distribution and abundance. To do so requires reliable, fine-scale biological and environmental data from sensitive species and ecoregions [14].

Mountainous regions provide exceptional opportunities to quantify effects of contemporary climate change on wildlife species and their habitats. First, mountainous regions, particularly those at high latitudes, have experienced some of the globe’s greatest temperature increases ([15,16] but see [17,18]) and may be expected to experience more warming in the future than other landscapes [19]. This trend is especially true in areas near the 0°C isopleth, where melting of snow creates a positive feedback loop of warming due to decreased albedo [17]. Second, montane species are among those most susceptible to the effects of contemporary climate change (e.g., [13,19,20,21]) because of dispersal challenges and because their distributions are strongly constrained by climate (e.g., [22]). Many high-elevation species are expected to respond to climate change with large range contractions [23], and many have already done so (e.g., [10,13,24]). These range shifts are generally characterized by contractions of the species’ lower-elevation limits (e.g., [15,25,26]). However, climate-change-mediated range shifts are not always upslope [27] and in some cases include significant range expansion [28,29]. More broadly, mountains provide freshwater for ~2/3 of the world’s human population [30], as well as diverse recreational opportunities. Finally, nationally and globally protected areas are located disproportionately in mountainous regions [31], yet montane areas remain understudied [13] and dramatically under-instrumented.

Increasingly, climate-adaptation and wildlife-conservation efforts rely on research that identifies the mechanisms by which climate is changing species’ distribution, abundance, phenology, and behavior. This ideal, while challenging, is sought precisely because this information is needed to inform conservation efforts, climate-adaptation management actions, as well as process-based models. It is challenging because *full* mechanistic understanding would include information on relevant macro- and microclimates, sympatric species, and the target species’ physiology, demography, behavior, and fine-scale distribution through time [32]. In a recent worldwide meta-analysis investigating mechanisms by which climate change has apparently acted, Cahill et al. [1] suggested that very few studies provided compelling evidence of proximate causal relationship between climate and local extinctions (*n* = 7 species) or abundance (*n* = 7 different species). They found that changing interspecific interactions–especially those leading to reduced food availability–were the most frequently demonstrated cause of extinctions and declines, more so than limited tolerances to high temperatures [1]. These and other authors have cited the need for more research investigating *how* and *why* climate is affecting species (e.g., [1,32,33]). Here, we measure proxies of American pika (*Ochotona princeps* Richardson) abundance, using several types of pika evidences (sightings, vocalizations, haypiles, and fecal piles), and do so across several hundred sites within a large protected area. Our study is also one of very few pika investigations with sample sizes large enough to develop and test models with withheld data.

Numerous characteristics of the American pika have been demonstrated (in manipulative experiments) and suggested (in correlative studies across space and time) to indicate that the species’ distribution, abundance, and fitness are strongly determined by climate. In the late 1960s and early 1970s, observations of pikas’ behavioral thermoregulation [34] and experimental restrictions of those behaviors [35] compellingly demonstrated that hyperthermia and death could occur at moderate (25.5–29.4°C) ambient temperatures. They found that pika basal metabolic rate is high (143% of predicted body-mass-specific value) and thermal conductance is low (101–53% of predicted values; [36]). As a result, resting body temperature of *O*. *princeps* is high (mean = 40.1°C) and upper lethal temperature is relatively low (mean = 43.1°C; [35]), leaving a narrow 3C° difference between the two. Using hundreds of museum records of *O*. *princeps*, Hafner [37,38] proposed the species as a biogeographic indicator of cool, mesic, rocky habitat. He derived an equation for “pika-equivalent elevation,” which predicts the minimum elevation of pika occupancy at a site, given its latitude and longitude: *E*(in m) = 14087 –(56.6)*°N– 82.9*°W.

In re-surveys of pikas across 25 sites with historical records of *O*. *princeps*, Beever et al. [39] found that climatic factors outweighed the predictive ability of biogeographic and proximate anthropogenic factors in explaining the pattern of loss of pikas from 24% of the sites. Grayson [40] placed those losses within a deeper historical context, by quantifying the increase in minimum elevation of pika records across the Great Basin through paleohistory. Beever et al. [41,42,43] found that climatic factors (e.g., average summer temperature, frequency of acute cold stress) appear to play increasingly important roles in continuing pika extirpations in the Basin, and that rates and drivers of extirpations, upslope retraction, and abundance have varied over time scales of less than a decade. Additionally, aspects of vegetation cover have also been shown to correlate with pika distribution and indices of pika abundance (e.g., [43,44,45,46]). Suitable habitat in the southern portion of the species’ range has been repeatedly forecasted to be largely eliminated under future climate conditions [47,48,49]. However, research in locations with physically complex habitats that provide climatic microrefugia (e.g., low-elevation lava flows and moss-covered, heavily forested gorges), has shown that the species’ status and trend in such habitats are more robust than expected, considering the region’s macroclimate [50,51,52,53,54]. To date, occupancy rates and apparent trends from re-surveys suggest that *O*. *princeps’* status is more favorable in the Sierra Nevada and Rocky Mountain mainlands (e.g., [55,56]) than in more-isolated areas [42,57].

Research over the last two decades on *O*. *princeps* has collectively shown the heterogeneity and nuance with which climate can act on the distribution and occupancy patterns of mountain-dwelling animals. For example, re-surveys of locations of historic pika records in the interior Great Basin have demonstrated loss of pikas from >44% of those locations [42,57]. In contrast, at higher-elevation sites in the southern Rocky Mountains, Erb et al. [56] reported that pikas persisted at 66 of 70 sites (5.7% loss). Similarly, across all of California, Stewart et al. [58] reported that pikas persisted at 57 of 67 sites (14.9% loss) at which pikas were reported in the 19^th^ and 20^th^ centuries. Research at various spatial resolutions has found both persistence and occupancy of *O*. *princeps* to reflect varying combinations of microclimatic, biogeographic, and vegetative predictor variables (e.g., [43,45,52]). For *O*. *princeps* and for all other animals, understanding the degree to which this heterogeneity in a species’ response to climate reflects differences in climate, landscape context, or the species’ genetic and epigenetic responses presents important opportunities for research.

Although occupancy has been the dominant focus for previous research on both pikas and other montane mammals, as is the case with all species, climate change will affect population abundances and community compositions before generating local extinctions. In terms of abundance of *O*. *princeps*, analyses from the hydrographic Great Basin have shown that the factors governing the patterns of pika abundance differed dramatically in surveys less than a decade apart, and that measures of climatic water-balance predicted pika density more strongly than did temperature metrics, in 2000s surveys [43]. Near Mt. Hood in the Cascade Range of Oregon, pika density was best predicted by species richness of plants and percent moss cover on the talus [59]. Two studies from the Rocky Mountains have used density of fecal-pellet piles (latrines) as proxies for pika abundance. In the southern Rocky Mountains, latrine density was best predicted by talus-patch area, followed by the diversity and relative cover of forbs [45]. In two mountain ranges in northwestern Wyoming, latrine density: was highest at middle elevations, increased linearly as forage availability increased, and increased with greater frequency of temperatures conducive to plant growth; however, pika density in this northern-range domain did not appear to support summer-heat hypotheses [60].

**Fig 1:**
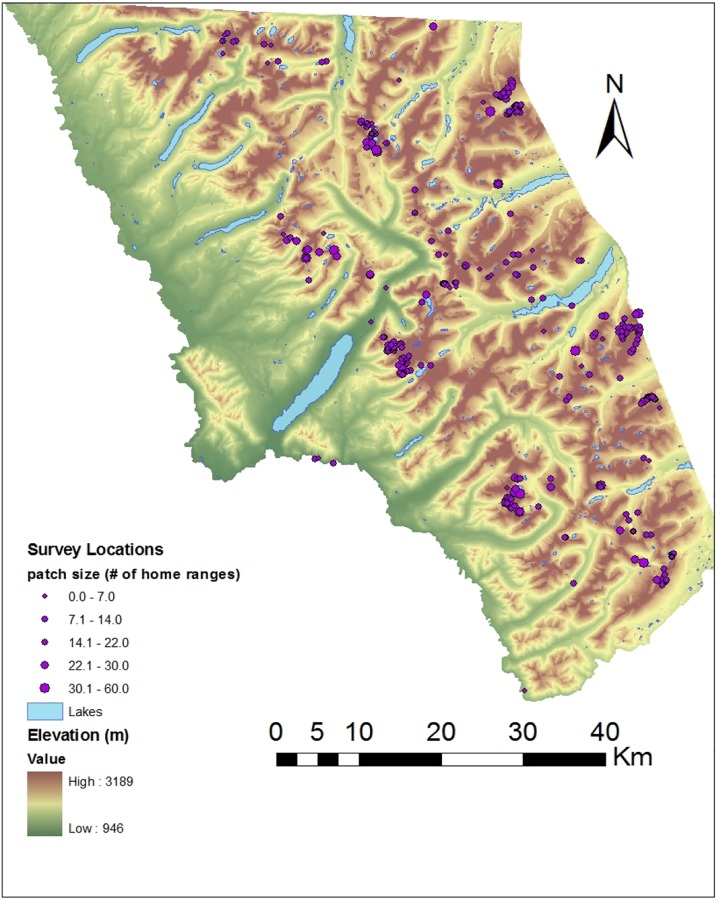
American pika survey locations in Glacier National Park, Montana, USA Sites (*N* = 314) at which we conducted pika surveys during 2007–2009, within the 405,000-ha Glacier National Park, Montana, north-central USA. The park is presented as a digital-elevation map; brown tones correspond to higher elevations. The diameter of each purple circle reflects the number of pika home ranges (HR) searched during that site’s survey.

Although there is strong evidence for climate-driven changes in the distribution of American pikas in some portions of the geographic range, comparatively less research has investigated *O*. *princeps* at the species’ leading, northern (range) edge, where cold stress and persistent snowpack may play increased roles in limiting distribution, such that north-facing slopes and the highest elevations may have lower pika abundance. If a shifting climate is indeed driving extirpations of American pika populations in parts of the species’ range, the northern Rocky Mountains may be one of the species’ final refugia in the U.S. ([47,48], but see [54] for microrefugia-driven persistence in complex microhabitats). Additionally, very few studies have examined pika abundance across a large network of sites (i.e., [43,45,56,59,60] each of which involved <100 sites), and those with relatively large sample sizes span entire eco-regions and only examine occupancy (e.g., [61]), not abundance. Here, we examine patterns and determinants of pika abundance across Glacier National Park (GNP), Montana, north-central USA (Fig 1), during 2007–2009 inclusive. Our objectives were to: 1) comprehensively identify pika habitat, testing the ability of remotely sensed data to predict pika-appropriate talus habitat; and 2) identify climate- and habitat-related predictors of longer-term and current pika occupation and abundance. Whereas current evidence (“Current Occupants”, described below) reflected the current year’s snapshot of pika abundance, longer-term evidences also included old haypiles and old fecal pellets, thus indexing patterns of abundance across the past several years to decades. This longer-term integration acknowledges the extinction-recolonization process of *O*. *princeps’* metapopulation dynamics within and across years. Results of these analyses will inform future monitoring efforts for *O*. *princeps* and other montane-ecosystem animals.

### Study area

Glacier National Park (GNP) sits roughly between 48–49°N latitude and 113–114°W longitude, and talus habitat that is physically appropriate for pikas occurs between 1050–3000 m elevation, and as identified by this study, covers ~2,800 ha. This provides an elevation range roughly analogous to that of earlier studies of *O*. *princeps*’ relationships to climate (e.g., [35,39,41]), although at latitudes 5 to 12 degrees farther north. The protected-areas complex that includes GNP (USA) and Waterton National Park (Canada) is often called “The Crown of the Continent”. It contains a unique biota and position within the Northern Rockies wilderness corridor, and represents the eastern edge of the area of highest ecological integrity within the contiguous USA [62]. The shrinking of GNP’s namesake alpine glaciers has been well documented (e.g., [63]) and has served as widely recognized evidence of climate warming.

## Materials and Methods

### Focal organism

The American pika is a model organism for testing hypotheses regarding climatic effects on species presence and abundance. It is a medium-sized (125–200 g) lagomorph that lives up to 7 years (mean life expectancy ~ 3 yrs), and is typically found in mountainous areas of western North America, from British Columbia and Alberta south to California across to New Mexico [64]. American pikas are most active on the surface during diurnal and crepuscular hours and obligately inhabit talus and other contexts with broken rocks 0.2–1.0 m in diameter [65]. Individuals exhibit a preference for the largest rocks for their haypiles, which are also their centers of activity [65,66]. Pikas’ obligate relationship to talus is noteworthy because, unlike habitats preferred by most species, the amount and spatial distribution of this habitat has been relatively unchanged for centuries. Without the effects of habitat modification, shrinkage, and fragmentation, the effects of climate change are easier to isolate [42,67].

*O*. *princeps* individuals are central-place foragers and territorial. They do not hibernate, and in regions without year-round forage availability, harvest vegetation during the summer and early fall to create one or more haypiles that partially meet their winter caloric needs [68] (see [69,70] for exceptions). The foraging radius extends 16–26 m from their haypile, of which the central (or “interior”) 10–15 m is a territory defended from other pikas [64].

Although pikas are considered perhaps the classic mammalian model for metapopulation dynamics and their associated extinction-recolonization processes, patch-level occupancy can be comparatively stable in pikas relative to species such as arvicolines, partly because pikas are highly philopatric and most movements within a season are <0.3–1 km [64,71]. Despite these tendencies, genetic analyses suggest that rare longer-distance dispersal events do occasionally happen and that genetic neighborhoods can extend as far as 4.2 km, although dispersal is more limited in hotter and drier contexts [72,63,73,74].

At the individual-territory scale, however, turnover can be extensive, because it can result not only from death or emigration from the patch, but also emigration to a different location in the patch. For example, turnover in Jeffress et al. (2013), as measured by re-visits to 265 of 1172 originally sampled 24-m-diameter circles (each approximating one home range) in the previous year, ranged from 0.49–0.74 across eight U.S. national parks [61]. At a site in the eastern Sierra Nevada during 1988–1991 inclusive, an average of 34% (range = 15–62.5%) of resident adults disappeared each winter, although 96% of adult residents that were present in the population for multiple years remained on the same territory. Kruezer and Huntly (2003) also reported high estimates of over-winter losses of individual pikas from tagged populations [75]. In sum, low turnover does not necessarily imply short-distance dispersal, and rates of dispersal and gene flow can vary markedly across the geographic range of *O*. *princeps*.

### Site identification

We identified pika-appropriate talus habitat by conducting visual scans from summits and ridges that allowed observations of valleys and nearby (i.e., within 7.0 km) mountainsides in the park, with the goal of observing every major valley in GNP, although a few of the most-remote valleys went without surveillance. From these vantage points, we identified talus deposits with extents of ≥1000 m^2^ and outlined them on 7.5-minute topographic maps. Patches <1000 m^2^ (i.e., 31.6 m x 31.6 m, which generally translates to 1–2 home ranges) were not included because consistent and accurate identification of patches this small was difficult from our remote vantage points. Furthermore, the vast majority of pika-appropriate habitat in GNP occurs in larger patches: ≤3 such small patches were observed and excluded from our surveys among all the patches identified, and each in a precarious–to-reach location. During 2007–2009, we identified 411 such talus patches, of which 314 were surveyed during the three-year study. The patch outlines and positions were estimated from our distant vantage points, whereas the patch area was estimated during site surveys because we were interested in the pika-appropriate area within a heterogeneous patch and not simply the area within the patch perimeter. The observed talus patches were then digitized into ArcGIS 9.3 [76]. Because we visually scanned the vast majority of the areas above tree-line in the park, we estimate that we identified at least 75% of the talus habitat in the park and surveyed >50% of the park’s total talus area for pikas. We considered patches distinct sites if separated from other such patches by either 100 m distance or by a significant dispersal barrier, such as a cliff or river. Finally, we compared the locations of talus patches that we observed to talus identified by remote sensing [77] to assess the accuracy of GIS-based talus identification.

### Site surveys

We conducted pika habitat identification and monitoring in GNP, with permission from the National Park Service, during 12 June– 23 August 2007, 1 June– 30 September 2008, and 11 June– 17 September 2009. Surveys began each season following sufficient snowmelt; across the season, they occurred on Julian date 205 ± 29.1 (25 July; mean ± 1 SD). Pikas are typically easy (*p* usually > 0.90) to detect (e.g., [42,51,56]), and we assume that to be the case in GNP because: 1) individuals in GNP generally construct and defend obvious haypiles; and 2) individuals use vocalizations both for conspecific attraction and for territorial announcement/defense, so these calls are often given when surveyors enter a talus patch. Although detectability can vary between regions and locations (e.g., lower at some anomalously low-elevation sites, described in [57]), and observer variability can influence results, our field crew received training and conducted trials to measure these factors. In GNP, repeated surveys by each member of the 2009 field crew revealed that the identity of the individual was not a significant predictor (multiple linear regression, F-test, p >> 0.05) of the number and type of pika signs observed nor home ranges estimated, with the exception of one individual who overestimated vocalizations and HRs [78].

#### Survey methods

Many aspects of the survey methods adhered closely to those of [79]. Surveys were conducted in teams of two observers. Each observer surveyed different, non-overlapping, sections of the talus patch. Observers traversed the patch in parallel, keeping at least 30 m between them. Observers traveled in an irregular zig-zag fashion, focusing on the places most likely to contain pika sign, such as near the largest boulders and near the talus/meadow interface [64]. Teams reconvened for the vegetation transect after searching for a maximum of 30 minutes or once the entire patch was searched. Multiple 30-min surveys were conducted if the patch was too large to be adequately covered in just one survey. Each 30-min survey constituted a site within a patch. To avoid pseudoreplication, we only included one site survey per patch in our analyses, selecting the site at which vegetation data were collected. If multiple sites within a patch included vegetation data, we randomly selected one of them. Latitude, longitude, and elevation were recorded at the first haypile identified during each site survey, using a Garmin 76Map CSx GPS unit (3–7 m accuracy). If no haypile was found, the GPS location and elevation were taken roughly in the center of the site. Flat sites (<5% slope gradient) were considered to have a southern aspect because they receive full sunlight. Surveys were not conducted during precipitation events.

#### Site size, isolation, and talus depth

We estimated patch size during surveys by tallying the number of potential pika home ranges available. Specifically, pika home ranges (HRs) were defined as non-overlapping 20-m-radius circles of pika-appropriate talus, following nearest-neighbor distances reported for *O*. *princeps* in [64] and extensive *pers*. *obs*. within GNP by LMH. Although HR size can vary between geographically distant locations, we found no evidence of substantial variation within GNP. We excluded from our counts talus areas that did not contain at least one >0.2 m-diameter rock. We indexed patch isolation in two ways: 1) by estimating the number of other talus patches visible from the site (e.g., on a mountain-side across the valley) and 2) by estimating the total number of home ranges within all those patches: 1–10, 10–30, or >30. We recorded the measurable depth of the deepest crevices within the talus as a metric of ‘insulation’ or the potential for pikas to escape surface temperatures and predators. We used four talus-depth categories (m): <0.5, 0.5–1.0, 1.0–1.5, and >1.5. We also recorded maximum rock diameter (in m, along the rock’s longest axis), within 1 m of each pika sign (see the following paragraph).

#### Site occupancy and pika abundance

Our goal was to develop a rapid approach for classifiying site occupancy and relative pika abundance. We did this by identifying multiple types of pika signs during surveys, including visual and aural detection of individuals, number of fresh and old haypiles discovered, and number of fresh and old piles of fecal pellets (hereafter, “scats”) discovered. To quantify the extent of habitat that was currently being (or had recently been) used by pikas, we tallied the number of non-overlapping pika HRs that contained ≥1 evidence of pika site use (***ID***). This estimate included both fresh and old evidences, and thus reflected longer-term evidence of occupancy. Multiple evidences (of the same or different type of sign) within a HR thus did not increase ID; ID was either 0 or 1 in each HR. We sought to minimize the error associated with estimating numbers of home ranges by: 1) using (20-m) diameters on the lower end of nearest-neighbor distances reported for *O*. *princeps* (14–34 m: [64]), because inaccuracy and variability both increase as distance increases, using ocular estimation; and 2) administering multiple, intensive field-crew training sessions–after these, we found consistency among our field crew in making these HR estimates (difference between individuals using multiple linear regression, F-test p >> 0.05 [78]). Improved accuracy could potentially be achieved by use of laser rangefinders to estimate distance of talus-patch dimensions (*sensu* [57]) or by using remotely sensed data, as in [58], although the latter can be extremely inaccurate, as quantified in this study, and suffers from not accounting for talus that is not pika-appropriate (generally, when talus rock diameters are all <0.2 m or >1.0 m; [65]).

We estimated the number of pikas currently occupying a site by summing the number of home ranges containing at least one fresh sign (i.e., fresh haypile, call, or sighting of an animal). Occupancy was either 0 or 1 in each HR. However, when extended across all HRs within a talus patch, this becomes a ratio that approximates abundance as the percent of a talus patch that is currently pika-occupied (i.e., ***Occupants/HR***). Fresh scat was not used to calculate Occupants because of the inconsistent ability of observers to unequivocally classify fecal pellets as old vs. fresh (e.g., see [80]). Although fresh haypiles are the best evidence of an ‘active’ or currently occupied territory, how ubiquitously they are employed varies dramatically across the species’ range [45,69,70], thus including calls and pika sightings as signs of current occupancy is a more comprehensive and widely applicable approach. We acknowledge that we may have occasionally encountered sightings and vocalizations from an animal outside its normal HR, and thus had rare occasions of double-counting. Conversely, we likely failed to detect some individuals (most commonly juveniles) that neither vocalized, created a haypile, nor were surface-active during our surveys. Given these challenges encountered with HR estimation and pika sign enumeration, and because home-range size may differ across fine-scale and especially across coarse-scale gradients, we consider our metrics proxy estimations of actual animal abundance. Although Occupant/HR and ID/HR represent fractions of the estimated number of home ranges in a patch (or a portion of a patch) with this year’s (current) or recent (longer-term) pika occupancy, respectively, these approximate the relative abundance of *O*. *princeps*. Because this method is intended to provide a rapid-assessment approach to determine relative abundance, we will refer to the Occupants/HR and ID/HR metrics simply as “current abundance”, “longer-term abundance”, or (when referring to both measures) “abundance”, throughout the rest of this study.

Search techniques and effort necessarily differ between direct and indirect signs. The detection of ‘indirect’ signs (haypiles and scat) requires focused searches at or below the surface of the talus, whereas the ‘direct’ (visual or aural) detection of individuals may occur at any time during the site survey. Search effort for indirect signs was quantified as the time spent specifically searching for hay and scat, and effort for direct signs as the total time spent at the survey site, which also includes time spent conducting vegetation transects.

#### Vegetation

Within each site, the total cover of each plant species was estimated using point-intercept (using a 1–2 cm diameter stick or pin as the point) sampling at each 1 m along line transects (sensu [81]). This is called the step-point intercept method because we paced out the transect rather than anchoring a piece of twine to the ground; it allows one to characterize plant cover within multiple layers of vegetation, and can be used to estimate plant height. We identified vegetation by life-form: graminoid, forb, shrub, tree, moss, and lichen. Pikas are generalist herbivores; however, pika foraging and haying are affected by the relative availability, size, and secondary chemistry of plants [68,82]. Much of the variability in secondary chemistry can be captured by identifying plants according to these broad classes. For example, forbs tend to be larger and defend themselves with toxic chemicals, whereas graminoids (grasses, sedges, and rushes) are relatively small and less toxic. Plant height was estimated to the nearest 0.5 inch and later converted to cm.

We sampled vegetation using a 24-m-diameter transect with its midpoint at a haypile or (in cases when we detected no haypiles within a site) at what appeared to be the best available habitat–near the biggest rocks, having the deepest crevices, and near at least moderate plant cover, often near the talus-meadow interface. In talus habitats, the fall line (steepest average slope gradient) often captures the most-dramatic vegetation gradient [79]. Thus, we oriented one of the three transects along this line to quantify the available forage resources. If the site was flat, the transect was oriented along the most (visually) apparent vegetation gradient. This first transect was crossed by two additional 24-m transects, such that the six endpoints of the ‘asterisk’ were each equally separated from adjacent endpoints by 60°.

#### Derivation of variables

Some pika-sign and habitat variables collected in the field were modified prior to habitat-selection analyses. For each site, we derived three novel response metrics from the suite of pika-sign data collected. These, in turn, reflected a) instantaneous (visual and vocal detections) and recent (fresh haypiles) abundance (i.e., Occupants/HR), b) longer-term patterns of abundance (any evidence of current or past occupancy; ID/HR), and c) the fraction of the habitat area that has ever been occupied that is currently occupied (i.e., Occupants/ID). The last variable had a lower *N*, because we removed from analysis all observations (*n* = 14) in which no HR had any evidence of *O*. *princeps* (to avoid division by zero). Predictor variables were derived as follows. To measure departure of a patch’s field-measured aspect from south-facing, we transformed aspect as follows: Adjusted aspect = |field-aspect– 180°|. We defined a binary variable ***Flat*** to be 1 if the slope gradient, as measured in the field, was <30%, and Flat = 0 otherwise. Although we predicted that abundance would exhibit a quadratic (‘peaked’) relationship to elevation, we also included elevation in models that included elevation^2^. Insulation reflected whether the talus depth was > 1.5m. We defined a seasonality variable as the number of days elapsed between 1 June and the survey date. Vegetation cover-types (e.g., Rock, Trees, Graminoids, Forbs, Moss) were each calculated as the proportion of total cover. Each suite of vegetation transects involved 72 cover measurements. Vegetation height was calculated as the mean height of plants from each cover-type per site.

#### Selection of sites for analyses

Analyses included only one survey per site at which a survey was conducted. When a site was surveyed multiple times (e.g., revisited within the same or a different year), we analyzed only the survey during which each data category was measured. When multiple surveys of a single site met those criteria, the survey to be analyzed was chosen randomly.

#### Data analyses

We compared a modest number of competing models (<38) chosen *a priori* that we hypothesized might best explain each of our response variables: long-term pika abundance (ID/HR), current abundance (Occupants/HR), and Occupants/ID. Each model suite was tailored to its corresponding response variable, based on earlier studies or field experience of the authors with *O*. *princeps* since 1994. These models were fitted to the exploratory subset of data (i.e., ‘original data’), about two-thirds of the observations randomly selected from the full data set. The same models fitted in the exploratory phase were later fitted to the withheld portion of the observations in the confirmatory phase to see if results were consistent. We used Akaike’s information criterion adjusted for small sample size (AIC_*c*_; [83]) to quantify the parsimony and fit of each model to each of the data sets. We used *P-*values associated with individual explanatory variables to assess the significance of each variable in a prescribed model. Inferences were made from the original (exploratory) data.

The variables HR, ID, and Occupants assume only non-negative integer values. We were interested in three ratios: ID/HR, Occupants/HR, and Occupants/ID. Because Occupants ≤ ID ≤ HR, these ratios are fractions that range between 0 and 1, so we used generalized linear models with a Binomial distribution and a logit link (logistic regression) to analyze them. Proc GENMOD in SAS 9.4 [84] was used for model fitting.

## Results and Discussion

### *A priori* identification of sampling sites

Among the initial patches that we examined in-person, most either did not have talus, or did not have rock diameters typically occupied by pikas (0.2–1.0 m;[65]). The GIS coverage primarily identified areas of scree instead of talus. Consequently, we thereafter identified patches for survey using binoculars at good vantage points (typically on ridges). Among our 411 patches identified *in situ*, only 167 (40.6%) had at least some overlap with the areas delimited as talus by remote sensing. The GIS coverage layer identified 708 patches and 11,576.8 ha of talus. In the portion of the park that we could observe from our surveillance from peaks and ridges (estimated to be ~75%, or 8682.6 ha, of the landscape identified as talus by the remote layer), we found that only 463.8 ha (~5%) in fact contained talus; thus, the remote layer had a ~95% false-positive rate. Additionally, the remote layer failed to identify 1607.15 ha of the 2070.93 ha of talus we observed; thus exhibiting a ~78% false-negative rate. Assuming we observed 75% of the total talus in GNP, we can estimate that there are ~548 patches and ~2761.2 ha of talus, park-wide.

Several phenomena accounted for the discrepancy between patches classified in the remotely-sensed surficial geology layer as “talus” and patches identified from ridge surveillance and subsequent at-patch assessment, highlighting limitations of using remote imagery to identify micro-habitat features, such as the size of individual rocks, which are critical for saxicolous animals such as the American pika. First, and most significant, the remotely-sensed layer was ineffective at properly identifying talus patches, as evidenced by its 95% false-positive and 78% false-negative rates. Although the majority of the false positives occurred on landscapes covered in scree (rocks too small to be pika-appropriate talus) or bedrock, the high rate of false negatives indicates broader inaccuracies in the imagery and landscape-identification algorithms. Second, several talus patches were found in unexpected places. This included talus patches surrounded by trees (and thus not visible from remote imagery), which were revealed only by wildfires (e.g., Divide Mountain) or when survey crews walked near those patches under the forest canopy. Atypical places also included rockpiles derived from man-made structures (as also found by Manning and Hagar [85] in the Cascade Range) such as the rock foundation for train tracks or a road (e.g., Red Rock Point site on Going-To-The-Sun Road). Third, although our ridge and peak surveys for talus allowed us to see most of the basins and cliff-bottoms in GNP, certain constraints prevented this method from identifying all potential talus patches in the park. In some instances, the view was obscured by clouds or smoke when observations were made. Furthermore, topography in GNP constitutes some of the steepest, cliff-ridden terrain across the geographic range of *O*. *princeps*. Thus, some basins, peaks, and ridges were obscured from view or simply unreachable in a safe manner, using ground-based surveys. Since ~2014, improvements in the resolution of publicly available remote imagery have, in some cases, allowed correct identification of pika-appropriate talus patches up to 80–90% of the time (EAB, *pers*. *obs*.)

### Site occupancy and pika abundance

**Fig 2:**
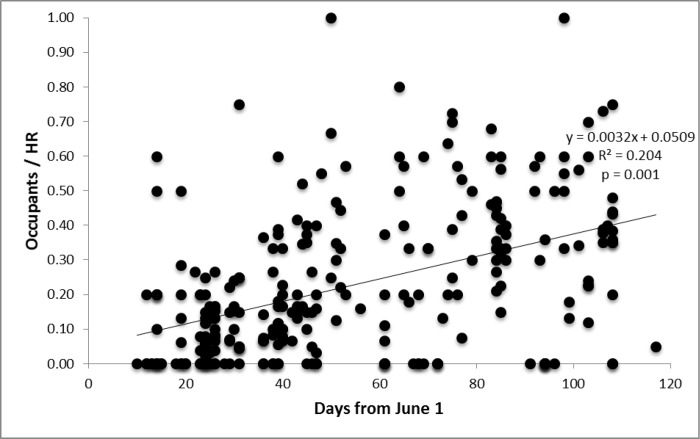
Short-term pika abundance increases as the summer progresses. Occupants per home range in relation to survey date at pika sites in Glacier National Park, Montana, USA (*N* = 277).

Across the three summers, surveyors hiked >16,000 km and conducted 360 surveys: 43 in 2007, 148 in 2008, and 169 in 2009. Some of the surveys (*N* = 46) were site re-visits, while 314 surveys (Fig 1) covered unique areas of potential pika habitat. Among the 46 re-visit surveys, 11 were re-surveys during the same day to assess observer bias and detectability. Among these, 10 recorded the same pika occupancy status (occupied), and one survey detected pikas at the site in the morning, but not in the early afternoon of the same day. Three re-visit surveys were conducted later during the same summer as the original survey; each of these observed the same occupancy status (occupied). Thirty-one re-visit surveys occurred across years, wherein we would expect that the assumption of population closure to be possibly violated. Among these, 21 recorded the same pika-occupancy status on both visits (19 occupied, 2 unoccupied), whereas 2 switched from unoccupied to occupied and 8 switched to unoccupied. Nineteen of the 314 unique surveys were incidental encounters of pika sign, which were not included in analyses. Across the remaining 295 surveys, we detected some evidence of pika occupancy (old, current, or both) in 274 surveys (92.9%), and evidence of current pika occupancy in 235 surveys (79.7%). Mean Occupants/ID decreased with each successive year of the study (mean ± 1 SE: 2007: 0.698 ± 0.091; 2008: 0.516 ± 0.032; 2009: 0.443 ± 0.024); however, this difference did not reflect inter-annual differences in Julian date of sampling. Within each year, Occupants/HR increased linearly as the season progressed (Occupants/HR = 0.05 + 0.0032*(# days after June 1); *r*^2^ = 0.20; p = 0.001; Fig 2). Across all three years, surveys that started during midday (1100–1500) exhibited 29.5% lower Occupants/HR than surveys conducted at other times.

GNP is home to a relatively large pika population, for which our study can provide a general size estimate. We surveyed 5065 potential home ranges and directly observed 1130 current occupants. Since we estimate 2761.2 ha of talus park-wide, we can also estimate 21,914.3 potential home ranges (assuming 0.126 ha per home range as an average) in the park. Extrapolating our park-wide averages of 23% Occupants/HR and 45% ID/HR as the lower and upper bounds gives a first approximation of a range of pika population size of 5040–9861 individuals within GNP. This population approximation necessarily relies on assumptions regarding how much of the park we didn’t observe and that these unobserved regions would have similar talus coverage and pika abundance to those we observed and visited. However, we include it here because our abundance measurements, in which we searched all the potential home ranges in each talus patch that we surveyed, recording every pika sign that we found, and detailed talus identification allow us to make the most-informed population estimate of American pikas across a large geographical region that exists in the literature, to our knowledge.

Application of Hafner’s (1993) equation for “pika-equivalent elevation” to GNP shows its limitations at this location, and likely at more northerly latitudes, in general. The predicted minimum habitable elevation (in m) for *O*. *princeps* at the lowest point of pika detection (48.69° N, 113.82° W) in GNP is:

$$14,087\;m-56.6\times \left(48.69\right)-82.9\times \left(113.82\right)=1895.4\;m$$

Despite this, we detected 266 pikas across 85 patches below 1895.4 m. We found pikas, in 2009, as low as 1056 m at the Red Rock Point site, which is among the lowest elevations present in GNP. Although Simpson (2009) found that pikas at elevations <100 m in the Columbia River Gorge between Oregon and Washington were also occurring far below Hafner’s predicted elevation, Varner and Dearing (2014) showed that this partly reflected strong decoupling of microclimates under an insulative moss layer from the regional macroclimate [69,70]. The location of pikas at Red Rock Point similarly reflects a refugial microclimate with the cooler and more-moist conditions afforded by McDonald Creek. Despite the 2009 detections, comprehensive re-surveys at Red Rock Point on the morning of 11 September, 2016, by 3 surveyors only uncovered old pika pellets; our next-lowest pika detection in 2007–2009 was 338 m higher.

Season progression strongly influenced our measurements of short-term pika abundance, Occupants/HR (Fig 2). We hypothesize that this seasonal phenomenon reflects the high latitude of the study area and the resultant persistence of snow later into the summer. Thus, pikas in GNP experience a shorter foraging and hay-collecting season than pikas in the majority of their range, forcing them to be highly active, especially during late summer. These pikas are easier to observe in that season; for example, they travel farther from the talus-meadow interface while haying [82]. Additionally, more pikas typically exist later in the season simply because of the influx (i.e., dispersal and territory establishment) of newborns. Pikas living at latitudes comparable to GNP typically give birth in early June and wean their young in July [86,87]. Therefore, there are more pikas that have dispersed to available habitat, later in the season.

These seasonal activity and detectability effects related to survey timing may confound attempts to measure patterns of pika occupancy. Like others [34,35], we found that pika activity and detectability were lowest during surveys occurring in the middle of the day, when temperatures are highest (data herein, and [88]). In addition to avoiding midday surveys, researchers should be mindful of the potential impact of season, especially if signs of short-term occupancy (e.g., sightings, fresh haypiles) are the primary goal. Because we did not account for detectability of individuals in all of our surveys (other than during the repeat training surveys [78]), we acknowledge that our response variables are likely proportional to underlying abundance, contingent upon the abovementioned assumptions and caveats.

Summary data on topographic, vegetative, and weather conditions across patches, as well as summary measures related to pika evidences, appear in S1 Table. All survey data is available in the Excel file, S2 Table.

### Evaluation of multiple competing hypotheses regarding patterns of pika abundance

#### Longer-term abundance (ID/HR)

**Fig 3:**
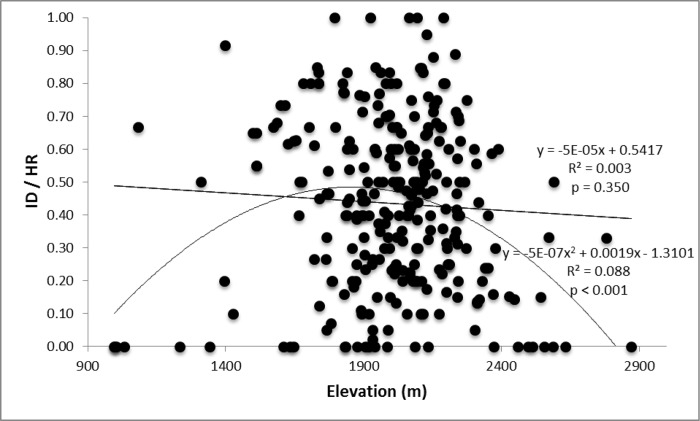
Longer-term pika abundance is greatest at intermediate elevations. Relationship between elevation (in m) of survey site and longer-term pika abundance (defined as ID divided by HR) in Glacier National Park, Montana, USA (*N* = 287, including 10 sites surveyed by EAB in 2011, aimed at increasing sample size at high elevations). Linear and quadratic fits are compared, here.

Across our suite of *a priori* models of longer-term pika abundance (ID/HR), a model including a site’s longitude, slope gradient, and elevation and elevation^2^ was clearly the top-ranked model using the original data (Table 1); all other models had ΔAIC_*c*_ ≥ 17.59. Using the withheld data, another model that included only abiotic predictors (talus depth > 1.5 m, longitude, and site elevation and elevation^2^) was top-ranked (Table 2), and the top model for the original data was marginally plausible (ΔAIC_*c*_ = 6.82). All other models had ΔAIC_*c*_ ≥ 23.85). Variable weights for the original data set highlighted the prominence of longitude, elevation and elevation^2^, and gradient above all other predictors–a pattern repeated in the withheld data, with the added importance of deep talus. Signs of the coefficients generally matched our predictions: ID/HR increased as one moved westward (towards the windward, higher-precipitation side; S1 Fig) or up in elevation to about 2300 m, and decreased as slope gradient became steeper (S2 Fig). This suggests that over the longer term, pika abundance is highest at intermediate elevations; ID/HR decreased at sites above 2300 m (Fig 3). The strength of this inference would be improved by more surveys at the lowest and highest elevations within GNP, but our reconnaissance suggests that few of such existing patches were excluded in this study, except perhaps some of the most-difficult-to-access, high-elevation sites. Although (as we hypothesized) large talus depths typically predicted higher ID/HR, this was not always true in the original data. Additionally, adjusted aspect was not particularly predictive of ID/HR in the regression analyses, but if site aspects were binned into four categories (North = 316 through 45°, East = 46 through 135°, South = 136 through 225°, and West = 226 through 315°), north-facing patches tended to have lowest levels of long-term abundance (17.1% lower mean ID/HR). Consistency of results between using the original vs. the withheld data was high, for both the long-term (ID/HR) and short-term (Occupants/HR) measures of pika abundance (Tables 1 and 2).

**Table 1:** Top models of short- and longer-term abundance of American pikas, using original data.

Response	Predictors	AIC_*c*_	ΔAIC_*c*_	*w*_*i*_
(a) Occupants / HR	i) forb ht, moss, elev, elev^2^	680.237	0.000	0.724
	ii) forb ht, moss, talus depth >1.5m, elev, elev^2^	682.199	1.962	0.260
	iii) forb ht, gradient, aspect, rock cover	687.853	7.616	0.016
	None (null)	758.745	78.508	0.000
(b) ID / HR	i) longitude, elev, elev^2^(-), gradient(-)	812.423	0.000	1.000
	None (null)	889.096	76.673	0.000
(c) Occupants / ID	i) forb ht, gradient, aspect, rock cover	475.531	0.000	1.000
	None (null)	538.011	62.480	0.000

Model results, using original data, including all models with ΔAIC_*c*_ < 10.5, and the null. Predictors were positively correlated to the response, unless otherwise indicated (-). The following abbreviations were used: “*w*_*i*_” = model weight, “elev” = elevation, “forb ht” = mean forb height.

**Table 2:** Top models of short- and longer-term abundance of American pikas, using withheld data.

Response	Predictors	AIC_*c*_	ΔAIC_*c*_	w_*i*_
(a) Occupants / HR	i) forb ht, moss, elev, elev^2^	274.830	0.000	0.582
	ii) forb ht, moss, talus depth >1.5m, elev, elev^2^	275.681	0.851	0.380
	iii) forb height, talus depth >1.5m, longitude	280.733	5.903	0.030
	iv) forb ht, longitude, moss	283.461	8.632	0.008
	None (null)	352.639	77.809	0.000
(b) ID / HR	i) talus depth >1.5m, elev, elev^2^(-), longitude	339.064	0.000	0.968
	ii) longitude, elev, elev^2^(-), gradient(-)	345.882	6.818	0.032
	None (null)	409.068	70.004	0.000
(c) Occupants / ID	i) graminoid+forb, forb ht	209.408	0.000	0.552
	ii) forb ht, aspect	211.552	2.144	0.189
	iii) forb ht	213.000	3.592	0.092
	iv) forb ht, gradient(9+,1-)^a^, aspect	213.715	4.307	0.064
	v) gradient(9+,1-)^a^, forb ht	215.005	5.597	0.034
	vi) forb ht, talus depth >1.5m	215.190	5.782	0.031
	vii) forb ht, gradient(9+,1-)^a^, aspect, rock cover(2+,1-)^a^	216.048	6.640	0.020
	viii) forb ht, talus depth >1.5m, longitude	216.116	6.708	0.019
	None (null)	252.850	43.442	0.000

Highest-ranking models, using withheld data, showing all models with ΔAIC_*c*_ < 10.5, and the null. Predictors were positively correlated to the response, unless otherwise indicated (-). The following abbreviations were used: “*w*_*i*_” = model weight, “elev” = elevation, “forb ht” = mean forb height.

^a^These predictor variables exhibited inconsistent relationships with the corresponding response variable across the suite of models that we chose. The notation indicates a positive (+) or negative (-) correlation and the preceding number is how many models exhibited that sign of the coefficient.

In our models, elevation was a strong predictor of longer-term pika abundance, ID/HR. Pika abundance tended to increase with elevation until ~1900 m, then remained relatively flat until ~2300 m, above which abundance decreased (Fig 3). Increased pika abundance with elevation is not surprising. Higher, cooler sites may provide pikas more time to be active feeding and haying during the day, thus increasing their fecundity. In the Great Basin, pika site extirpations were predicted by higher mean temperatures during summer [41]. Although based on a small sample size, the sharp drop-off in ID/HR exhibited by 10 of the 13 sites above 2400 m (Fig 3) may reflect a combination of several factors. First, talus above 2400 m in GNP is typically snow-free only during the second half of July, August, and most of September (LMH, *pers*. *obs*.), though snow persistence increases as one moves from the drier (eastern) edge of GNP to the higher-precipitation (western) edge. This provides an extremely short time-frame for pika reproduction, haying, and vegetation growth; typically one month less than at sites below 2000 m (LMH, *pers*. *obs*.). The lower value of ID/HR on north-facing sites may also reflect shorter snow-free periods. Timing and predictability of spring snowmelt are strong predictors of pika fatalities in *O*. *princeps* [89], and earlier spring conditions and plant phenology were associated with higher *O*. *collaris* adult survival over the following winter [90]. Second, high-elevation sites are exposed to significant wind-shear all year [91]. These winds may remove snow-pack that serves as crucial insulation for pikas during cold winter spells. In support of this second mechanism, acute winter cold-stress has been found [41] to strongly predict pika extirpations in the Great Basin. Finally, vegetation cover at sites >2400 m is nearly absent, at least on the eastern side of GNP; this has obvious nutritional and energetic consequences.

#### Short-term abundance (Occupants/HR and Occupants/ID)

**Fig 4:**
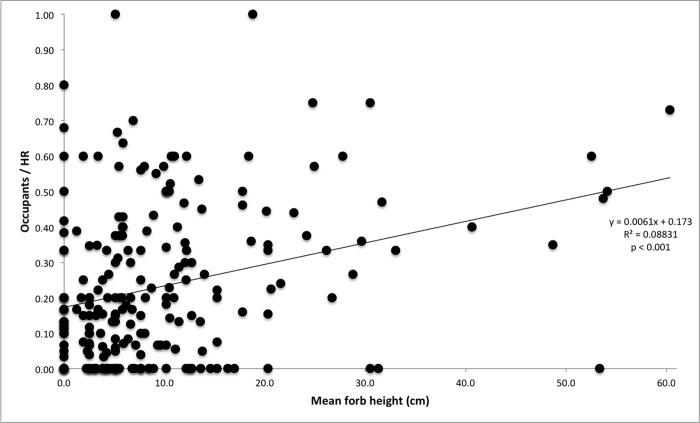
Short-term pika abundance is positively correlated to forb height. Positive relationship of mean forb height to occupants per home range at sites in Glacier National Park, Montana, USA (*N* = 223). Cover data were recorded along six, 12-m-long line-point transects radiating in 60° increments from one haypile per site.

Across our suite of *a priori* models of current abundance (Occupants/HR), average forb height, moss cover, and elevation and elevation^2^ were most predictive (Tables 1–3). They all had variable weights roughly four times larger than an index of insulation (i.e., nearby presence of insulative talus depth > 1.5 m). In particular, average forb height appeared in all of the 12 best-supported models, yet in none of the remaining models. For both the original and withheld data, the two most-plausible models contained that predictor (forb height) as well as percent moss cover, elevation, and elevation^2^; one also contained the index of insulation. Signs of coefficients for the most-predictive variables (moss cover, elevation and elevation^2^, and average forb height) were uniformly consistent across all models using both the original and withheld data. Specifically, as we hypothesized, short-term abundance increased with moss cover (S3 Fig), average forb height (Fig 4), and elevation; and exhibited a hump-shaped relationship to elevation^2^ (S4 Fig). Also as predicted, it consistently increased with more-westerly longitude and presence of very-deep talus.

**Table 3:** Top predictors of short and longer-term abundance of American pikas, using original data. For each of the three response variables, predictors are listed in order of descending *w*_*i*_/# of models.

Response	Predictors	*w*_*i*_	*w*_*i*_/# of models	# of models
(a) Occupants / HR	i) moss	0.984	0.141	7
	ii) elev, elev^2^	0.984	0.123	8
	iii) forb ht	1.000	0.083	12
(b) ID / HR	i) longitude	1.000	0.200	5
	ii) elev, elev^2^(-)	1.000	0.167	6
	iii) gradient(-)	1.000	0.111	9
(c) Occupants / ID	i) rock	1.000	0.333	3
	ii) forb height	1.000	0.125	8
	iii) aspect	1.000	0.111	9
	iv) gradient	1.000	0.100	10

Predictors with weight/number of models >0.03, using original data. Predictors were positively correlated to the response, unless otherwise indicated with “(-)”. The following abbreviations were used: “*w*_*i*_” = model weight, “elev” = elevation, “forb ht” = mean forb height.

Our approach of investigating patterns of both current occupancy and longer-term occupancy yielded noteworthy results. Specifically, other than elevation (which was a top predictor for both CO/HR and ID/HR and which covaries with several static and dynamic variables), the current year’s abundance was best predicted by two dynamic (biotic) variables (forb height and moss cover), whereas the proxy of longer-term pika abundance most strongly reflected more-static abiotic factors that apparently govern suitability for pikas across years. We encourage other researchers to replicate analogous investigations of this rarely employed approach across diverse other contexts.

Across our suite of *a priori* models regarding the Occupant/ID response variable, results differed significantly when using the original vs. the withheld data (Tables 1 and 2). With the withheld data, mean forb height and combined cover of graminoids and forbs were the only predictors in the top-ranking model, and the latter predictor appeared in each of the eight best-supported models but in none of the remaining models. In contrast to the short-term (Occupants/HR) and long-term measures of pika abundance (ID/HR) and perhaps intuitively given that it reflected a dividend of the two, Occupants/ID was best predicted by a mix of biotic and abiotic factors (Tables 1–3; S5 and S6 Figs). Two predictor variables, gradient and rock cover, exhibited inconsistent correlations with Occupant/ID; each showed a negative correlation in one model, but a positive one in the other models. Using the original data, one model including average forb height, slope gradient, aspect, and rock cover was clearly superior to all others, and across all models, graminoid and forb cover had markedly lower variable weight per model (0.00) it was used in, compared to analyses using the withheld data (Tables 1–3). Across all three response variables, ΔAIC_*c*_ for the null model ranged from 43.4 to 78.5, indicating that the predictor variables indeed predict CO/ID usefully.

Local vegetation provides additional insight into pika habitat preference (Tables 1–3, Fig 4). Our results suggest that, at least in GNP, habitats exhibiting robust forb growth are preferentially selected by pikas and/or may better support their survival in the longer term. Others have found similar positive correlations between pika site use and vegetation cover or quality (e.g., [44,45]). However, interpretation of the abundance-vegetation interaction is complicated by the pikas’ foraging relationship with the vegetation in their habitat and by the effects of microclimate on both plants and pikas (discussed in [52]). For example, it can be argued that the dominant vegetation cover represents those species remaining following harvest by *O*. *princeps* [92]. Pikas forage selectively on plants with higher caloric, protein, lipid, and water content [86,93]. Plant selection also occurs in a sequence that typically corresponds to the plant species’ seasonal phenology [82,94,95]. The presence of high-quality vegetation, such as forbs, may help pikas avoid patch- or site-level extirpations in portions of GNP, as may be the case in more southerly portions of their range [56].

We did not find a relationship between graminoid or forb cover and pika abundance; however, we did find that forb height is predictive of pika abundance. Plant height can vary dramatically even within species, and height indexes not only relative herbivory pressure (within a given plant species) but also shade and cover for *O*. *princeps* and other montane animals. Thus, taller forbs may reduce incoming solar radiation for foraging pikas, allowing for longer bouts of activity, especially on relatively hot days (e.g., [88]). Additionally, tall forbs may be indicative of relatively moist and resource-rich habitat with few competing herbivores.

#### Graminoid and forb exploratory analyses

Because several studies have found various versions of forb and graminoid cover to strongly predict pika occupancy or relative abundance [45,52,96] and because our results were not intuitive regarding the significance of forb height but not forb cover, we used our data in *post-hoc* analyses to explore the exact relationship of our three response variables (ID/HR, Occupants/HR, and Occupants/ID) to various mathematical combinations of forb (F) and graminoid (G) cover. These combinations included: F+G, F/G, and F-G. We chose these combinations to represent ametric of total herbaceous forage availability (F+G) and coarse indices of relative forb cover (F/G, F-G). For both CO/HR and ID/HR, for all twelve of our competing models (six, for each response) that contained graminoid and forb cover, the F/G variable provided the lowest deviance/DF. We used deviance/DF to compare fit because of differences in sample size across the four combinations due to zero denominators. When comparing AIC_*c*_ values among observations with graminoid cover >0%, F/G was the best predictor except that (forb-graminoid) performed 1.7 AIC_*c*_ units better than F/G in one model for CO/HR. Results were different, however, for CO/ID: deviance/DF was lowest for four of the six models using F/G, but lowest for the remaining two models using (F-G). More tellingly, using only observations for which graminoid cover was >0%, (F-G) had the lowest AIC_*c*_ value; this was >2 AIC_*c*_ units lower than that for F/G in 4 of the 6 models, and 1.83 units in a fifth model. The superior performance of the ratio F/G suggests either that the absolute cover of forbs (and graminoids) has less importance than the *relative* availability of forb-associated nutrients or that forbs tend to reflect more-mesic conditions than graminoids do [52].

As reviewed by Ray et al. [52], the importance of vegetation in explaining spatial patterns of pika distribution may reflect: a) the importance of forage quality for persistence of *O*. *princeps*; b) the fact that pikas influence composition and structure of the plant community; or c) microclimate driving plants and pikas in parallel fashion. Our initial modeling results indicated that forb and graminoid cover, individually or in combination, were not predictive of pika abundance in GNP; however, forb *height* (but not graminoid height) was predictive. These results do not necessarily help us rank the importance of the three explanations listed above. However, one plausible explanation may support factor b). GNP has a short growing season yet relatively high summer temperatures. Pikas inhabiting regions with extreme seasonality are expected to exhibit heightened caching selectivity, such that they directly consume graminoids and wait until late in the growing season to cache forbs, which might be expected to allow growth of taller forbs and produce higher (F/G) ratios. This selectivity may be enhanced when ambient temperatures are higher and foraging time is more restricted [97].

#### Future pika research

American pika population dynamics and responses to climate change will be most comprehensively revealed by pika surveys over longer time periods and large spatial extents. Although occupancy is a metric that can be applied at all areas across a species’ geographic range [98], abundance represents an earlier-stage response of wildlife to climate change (given that drops in abundance will always precede extirpations, except in catastrophes). Continuing research and monitoring on pikas in GNP will help illuminate whether 1) the monotonic decrease in abundance over this study’s three years, 2) the four times more-frequent change in site occupancy from occupied to unoccupied compared to from unoccupied to occupied, and 3) lack of pikas at the lowest-elevation (previously occupied) site for the last 1–2 years, constitute an ephemeral or more-permanent trend of pika abundance and distribution within GNP.

The baseline occupancy and abundance data presented here provide a unique opportunity for extensive meta-population research. Source-sink dynamics of the sites would be particularly interesting to explore as a complement to previous research [99,75]. The sites naturally vary substantially in area and isolation, and patch quality may be indexed by factors such as talus depth and extent, as well as vegetation cover. The intervening matrix clearly contains areas of varying suitability for pikas, but continuing research in landscape genomics should help clarify the degree to which various topographic features (e.g., cliffs, water bodies) and climatic aspects affect pika dispersal and survival, across GNP and other contexts.

GNP experienced numerous forest fires in the decade preceding and years following this research. The effects of forest fires on pika population dynamics provide another area for future research, as the effects may be strongly context-dependent (e.g., see [59]). Fire sometimes opens up previously forested talus patches. Pikas are positively associated with patches that allow them large viewsheds for predator surveillance [65]. Fires also stimulate herbaceous growth and may reduce predation pressure at previously marginal sites and lead to site colonization, re-colonization, and increased abundance of pikas over time (e.g., one site described by [97]). On the other hand, fires (especially at lower elevations) may be the vector by which non-native plants establish adjacent to taluses, which would commonly translate into lower-quality forage for a longer period of the year than if vegetation were native [100].

## Conclusions

In contrast to evidence of climate-mediated distributional shifts in *O*. *princeps* at the southern edge of the species’ distribution, pikas in GNP appear to be abundant across a broad range of locally available topographic and climatic contexts. In this leading-edge region, the areas from which pikas are temporarily or (in some locations) totally absent appear to primarily exist at the upper-elevation boundary of the species’ range, in the park’s highest-elevation areas, although the single lowest-elevation site that was pika-occupied during 2007–2009 appeared in September 2016 to have clearly lacked pikas for multiple years. We pioneered a method for measuring a proxy of pika abundance that includes indexing both short- and longer-term abundance. We found the clearly best-supported (top) model for the two metrics of short-term pika abundance included both vegetative and non-vegetative predictors; whereas the top model for our metric of longer-term pika abundance included static (abiotic) but not vegetative predictors.

## Supporting Information

S1 Table Pika survey summary statistics. Mean, SD, and sample size for each type of data collected to characterize pika habitats and weather conditions during surveys for *O*. *princeps* in Glacier National Park, Montana, USA, during 2007–2009. Cover data were recorded using six, 12-m step-point transects (following Herrick et al. 2009) radiating from one haypile per site, separated radially by 60 degrees (approximating an asterisk). Pika-sign variables reflect the number of each type of sign per survey conducted.
(PDF)

S2 Table Raw data. Excel file of the raw data collected in Glacier National Park, Montana, USA, during 2007–2009.
(XLSX)

S1 Fig Inverse relationship between longitude and longer-term pika abundance. Inverse relationship between longitude and the proportion of pika home ranges surveyed that contained at least one unequivocal pika sign (ID) at sites in Glacier National Park, Montana, USA (*N* = 277). The x-axis displays westerly longitudes on the left, easterly to the right. ID/HR can be interpreted as a measure of pika abundance over the long term.
(TIF)

S2 Fig Inverse relationship between site gradient and longer-term pika abundance. Inverse relationship of site gradient to proportion of home ranges surveyed that contained at least one unequivocal pika sign (ID) in Glacier National Park, Montana, USA (*N* = 277). ID/HR can be interpreted as a measure of pika abundance over the long term.
(TIF)

S3 Fig Positive relationship between moss cover and short-term pika abundance. Positive relationship of moss cover to number of pika occupants per home range at sites in Glacier National Park, Montana, USA (*N* = 225). Cover data were recorded using six, 12-m step-point transects (following Herrick et al. 2009) radiating from one haypile per site, separated radially by 60 degrees (approximating an *). Occupants/HR can be interpreted as the proportion of home-range-sized areas of habitat that are currently occupied.
(TIF)

S4 Fig Hump-shaped relationship between elevation and short-term pika abundance. The relationship between elevation (m) of survey site and number of pika occupants per home range at sites in Glacier National Park, Montana, USA (*N* = 287, including 10 sites surveyed by EAB in 2011, aimed at increasing sample size at high elevations). Occupants/HR can be interpreted as the proportion of home-range-sized areas of habitat that are currently occupied.
(TIF)

S5 Fig Positive relationship between forb height and short-term pika abundance. Positive relationship of mean forb height with number of pika occupants per home range containing any unequivocal pika sign (ID) in Glacier National Park, Montana, USA (*N* = 203). Cover data were recorded using six, 12-m step-point transects (following Herrick et al. 2009) radiating from one haypile per site, separated radially by 60 degrees (approximating an *). Occupants/ID can be interpreted as the proportion of home ranges with any pika sign (old or current) that was currently occupied by pikas.
(TIF)

S6 Fig Positive relationship between rock cover and short-term pika abundance. Positive relationship of rock cover with number of pika occupants per home range containing any unequivocal pika sign (ID) in Glacier National Park, Montana, USA (*N* = 205). Cover data were recorded using six, 12-m step-point transects (following Herrick et al. 2009) radiating from one haypile per site, separated radially by 60 degrees (approximating an *). Occupants/ID can be interpreted as the proportion of home ranges with any pika sign (old or current) that was currently occupied by pikas.
(TIF)
