# Supplementary material for: Predictors of Current and Longer-Term Patterns of Abundance of American Pikas (Ochotona princeps) across a Leading-Edge Protected Area
Source: PLoS One. 2016 Nov 30;11(11):e0167051. doi: 10.1371/journal.pone.0167051 (PMC5130250; doi:10.1371/journal.pone.0167051)
Supplement: S1 Table — Mean, SD, and sample size for each type of data collected to characterize pika habitats and weather conditions during surveys for O. princeps in Glacier National Park, Montana, USA, during 2007–2009. Cover data were recorded using six, 12-m step-point transects (following Herrick et al. 2009) radiating from one haypile per site, separated radially by 60 degrees (approximating an asterisk). Pika-sign variables reflect the number of each type of sign per survey conducted. (PDF) [file pone.0167051.s001.pdf]

**Variable**	**Mean**	**SD**	**N**
Environmental variables			
Elevation (m)	1990.3	271.4	303
Gradient (%)	21.2	10.0	303
Visible patches	2.7	2.4	290
Cloud cover (%)	41.3	33.2	299
Temperature (ºC)	18.2	6.3	301
Wind speed (km/hr)	14.9	9.4	303
Rock cover (%)	48.6	22.3	244
Lichen rock cover (%)	27.5	37.5	244
Forb cover (%)	7.9	8.0	244
Forb height (cm)	9.3	10.3	242
Grass cover (%)	4.3	5.3	244
Grass height (cm)	10.3	12.1	242
Shrub cover (%)	3.5	4.9	244
Shrub height (cm)	55.1	253.4	242
Tree cover (%)	1.3	3.0	244
Tree height (cm)	91.0	357.6	242
Moss cover (%)	2.5	4.9	244
Dead vegetation cover (%)	2.2	4.2	244
Dead wood cover (%)	3.0	4.9	244
Substrate soil (%)	7.3	9.1	244
Substrate rocky-soil (%)	13.9	13.5	244
Substrate rock (%)	71.4	20.7	244
Substrate snow (%)	1.7	6.4	244
Rocks < 1 m near sign	1.4	2.3	292
Rocks $1 < x < 2$ m near sign	1.9	2.5	292
Rocks > 2 m near sign	5.5	6.5	292
Pika-sign variables			
Sightings	0.84	1.71	294
Short calls	10.0	18.7	294
Long calls	0.18	0.57	294
Old hay	3.8	3.8	294
Old hay volume (dm^3)	127.1	236.4	211
Hay to veg. distance (m)	2.7	2.0	55
Fresh hay	1.2	2.4	294
Fresh hay volume (dm^3)	68.6	118.5	99
Scat	8.3	7.5	295
Total signs	24.3	29.0	295
Pika ID	7.4	5.7	295
Current Occupants	3.8	4.2	294
Home Ranges (HR)	17.0	9.5	295
Pika ID / HR	0.45	0.26	295
Occupants / HR	0.23	0.21	295
Occupants / ID	0.49	0.32	274
