# Supplementary figures and images for: Predictors of Current and Longer-Term Patterns of Abundance of American Pikas (Ochotona princeps) across a Leading-Edge Protected Area
Source: PLoS One. 2016 Nov 30;11(11):e0167051. doi: 10.1371/journal.pone.0167051 (PMC5130250; doi:10.1371/journal.pone.0167051)

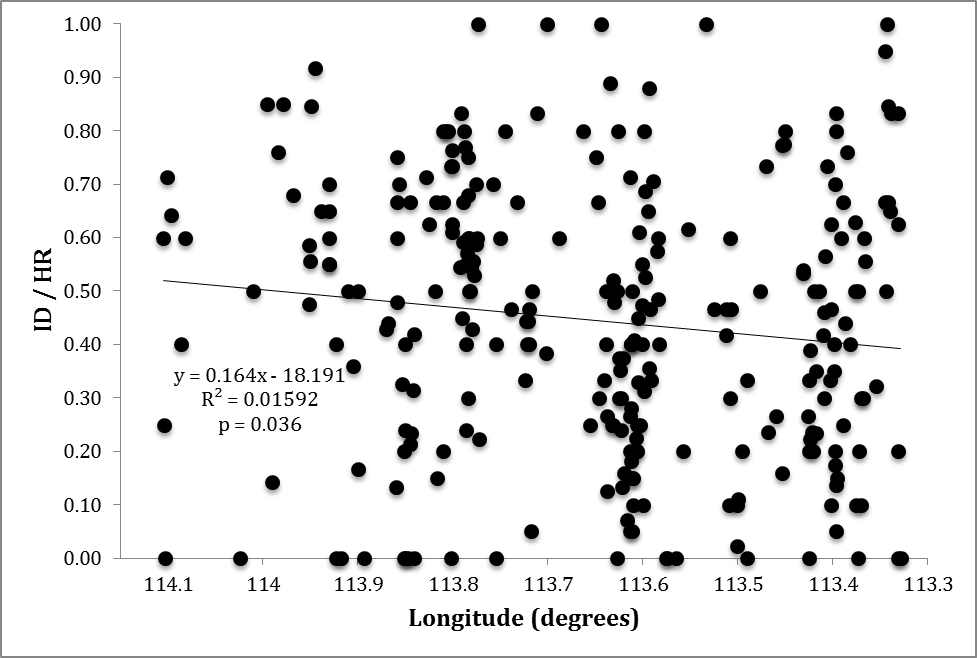

Supplement: S1 Fig — Inverse relationship between longitude and the proportion of pika home ranges surveyed that contained at least one unequivocal pika sign (ID) at sites in Glacier National Park, Montana, USA (N = 277). The x-axis displays westerly longitudes on the left, easterly to the right. ID/HR can be interpreted as a measure of pika abundance over the long term. (TIF) [file pone.0167051.s003.tif]

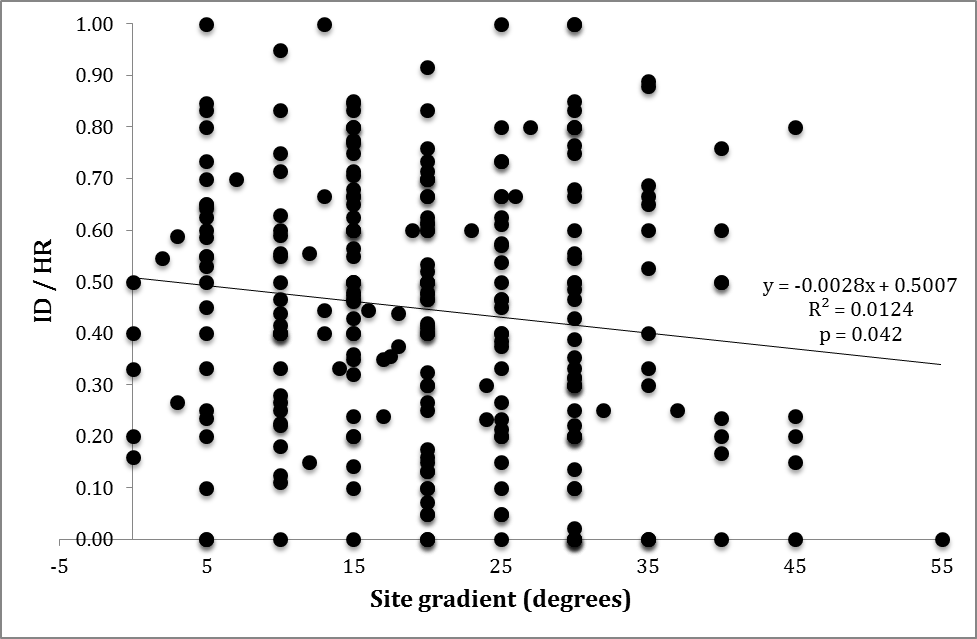

Supplement: S2 Fig — Inverse relationship of site gradient to proportion of home ranges surveyed that contained at least one unequivocal pika sign (ID) in Glacier National Park, Montana, USA (N = 277). ID/HR can be interpreted as a measure of pika abundance over the long term. (TIF) [file pone.0167051.s004.tif]

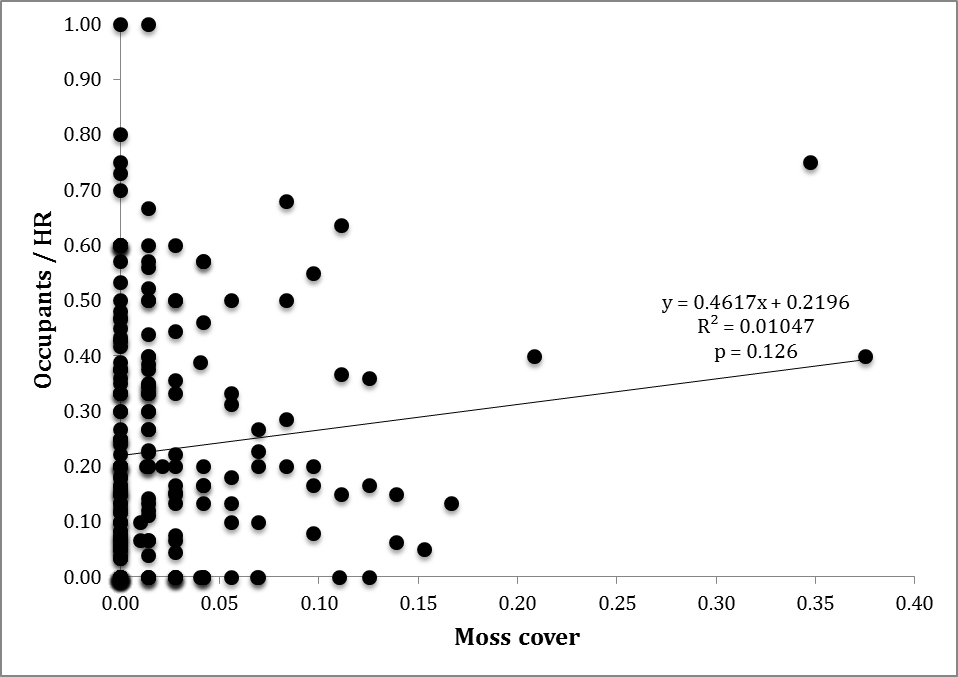

Supplement: S3 Fig — Positive relationship of moss cover to number of pika occupants per home range at sites in Glacier National Park, Montana, USA (N = 225). Cover data were recorded using six, 12-m step-point transects (following Herrick et al. 2009) radiating from one haypile per site, separated radially by 60 degrees (approximating an *). Occupants/HR can be interpreted as the proportion of home-range-sized areas of habitat that are currently occupied. (TIF) [file pone.0167051.s005.tif]

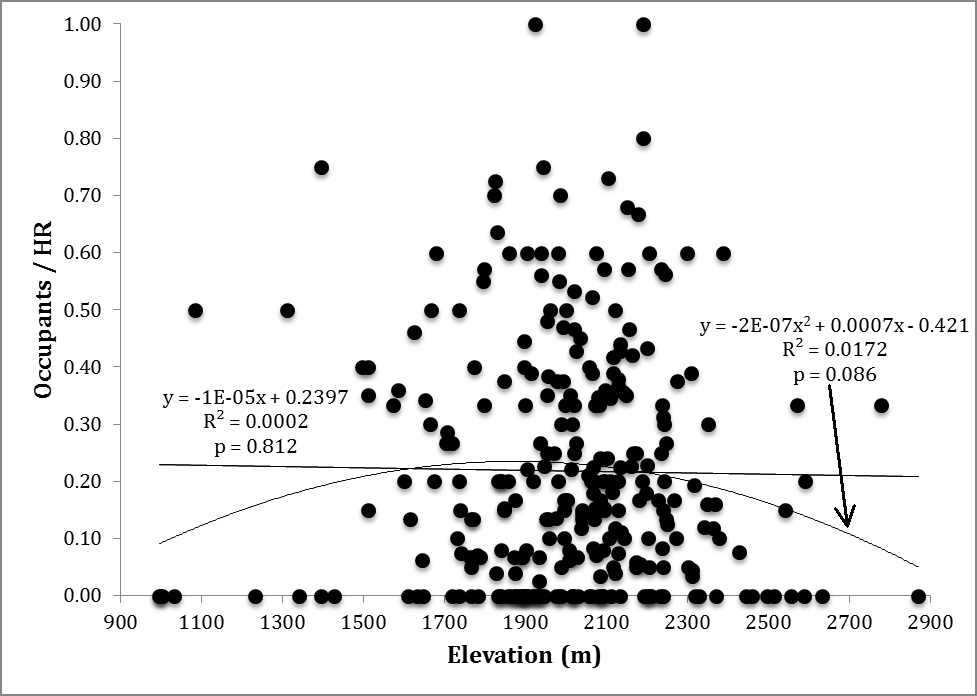

Supplement: S4 Fig — The relationship between elevation (m) of survey site and number of pika occupants per home range at sites in Glacier National Park, Montana, USA (N = 287, including 10 sites surveyed by EAB in 2011, aimed at increasing sample size at high elevations). Occupants/HR can be interpreted as the proportion of home-range-sized areas of habitat that are currently occupied. (TIF) [file pone.0167051.s006.tif]

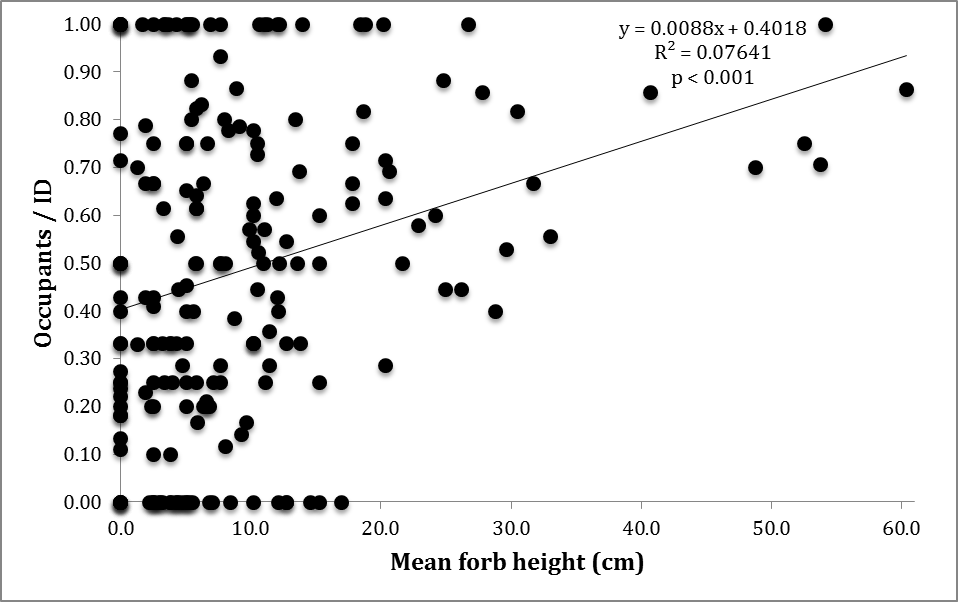

Supplement: S5 Fig — Positive relationship of mean forb height with number of pika occupants per home range containing any unequivocal pika sign (ID) in Glacier National Park, Montana, USA (N = 203). Cover data were recorded using six, 12-m step-point transects (following Herrick et al. 2009) radiating from one haypile per site, separated radially by 60 degrees (approximating an *). Occupants/ID can be interpreted as the proportion of home ranges with any pika sign (old or current) that was currently occupied by pikas. (TIF) [file pone.0167051.s007.tif]

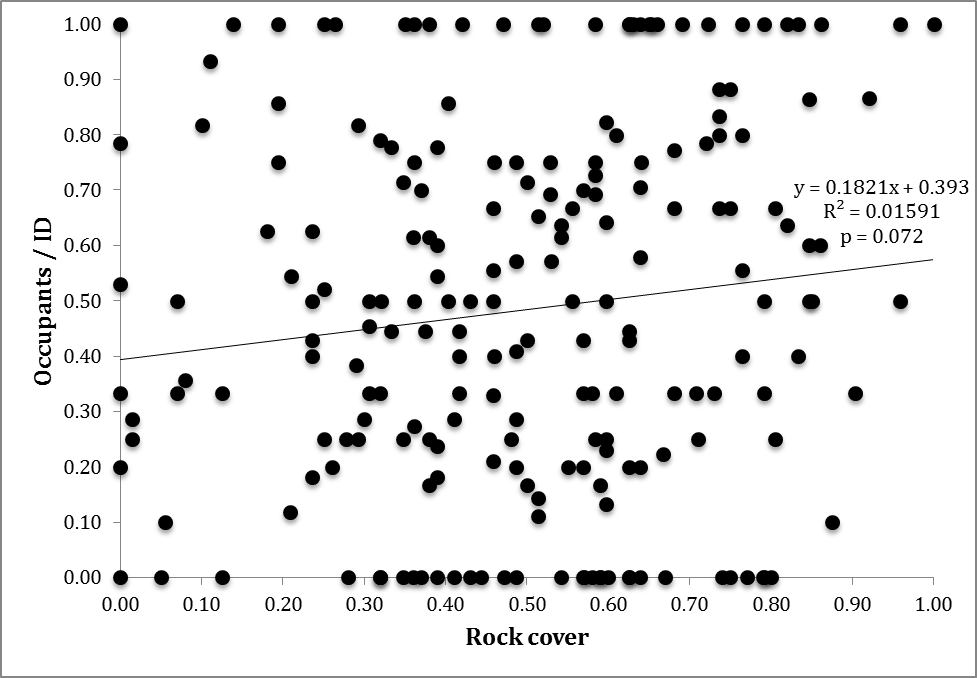

Supplement: S6 Fig — Positive relationship of rock cover with number of pika occupants per home range containing any unequivocal pika sign (ID) in Glacier National Park, Montana, USA (N = 205). Cover data were recorded using six, 12-m step-point transects (following Herrick et al. 2009) radiating from one haypile per site, separated radially by 60 degrees (approximating an *). Occupants/ID can be interpreted as the proportion of home ranges with any pika sign (old or current) that was currently occupied by pikas. (TIF) [file pone.0167051.s008.tif]
